# COVID-19 in Gansu Province, China: Characteristics, measures, and effects

**DOI:** 10.7189/jogh.11.03001

**Published:** 2021-01-13

**Authors:** Huanhuan Dong, Lufang Feng, Na Zhang, Zeshan Zhu, Xingrong Liu

**Affiliations:** 1 School of Public Health, Lanzhou University, Lanzhou, PR China; 2 Gansu Provincial Hospital Management Center (Gansu Provincial Family Planning Medicine Station), Lanzhou, PR China

Corona Virus Disease-2019 (COVID-19) infections have spread worldwide since it was first reported in December 2019 and is now a global pandemic. So far, China has been successful in preventing and controlling the spread of the disease. Although economically underdeveloped, the Gansu province of China also responded quickly and achieved excellent results in preventing and controlling the epidemic, with high recoveries and low mortality rates. This paper aims to discuss the measures adopted by the Gansu Province of China for COVID-19 prevention, treatment, and control and to provide reference for preventing, treating, and controlling the disease.

## STATUS OF COVID-19 IN GANSU PROVINCE

Gansu Province is an economically undeveloped area situated in the northwest of China. Over 26.4 million people live in the region, of which 3.1 million (11.6%) are more than 65 years old. The province is about 425 800 km^2^ and includes 14 states/cities namely: Lanzhou, Jiayuguan, Jinchang, Baiyin, Tianshui, Wuwei, Zhangye, Jiuquan, Pingliang, Qingyang, Dingxi, Longnan, Linxia Hui Autonomous Prefecture, and Gannan Tibetan Autonomous Prefecture. Lanzhou is the capital city of Gansu Province, with over 3.7 million people, whereas Tianshui is the second-largest city, with over 3.3 million people. Although Gansu Province does not border Hubei Province (which was the most hard-hit province in China), it also reported a considerable number of COVID-19 cases.

By November 3, 2020, China had reported a cumulative total of 86 087 confirmed cases, of which 392 were active, 81 061 cured, and 4634 died [1].

The epidemic was first reported in Gansu Province on January 23, 2020, and by March 14, 2020, all the 91 local confirmed cases had been concluded [2]. No case was reported in Wuwei, Jiuquan, and Jiayuguan, whereas Lanzhou and Tianshui reported more than 10 cases. As of November 3, 2020, 88 imported cases had been reported in Gansu province, of which 78 were cured, and 10 were treated in isolation in provincial designated hospitals [3].

## CHARACTERISTICS OF CONFIRMED COVID-19 CASES

Most confirmed cases were young adults, and this may be related to the economic activities of the population. Most young adults are either students or employed, and therefore exhibit high mobility. The youngest patient was one year old, whereas the oldest was 94 years, indicating that the novel coronavirus affected people of all ages. Notably, the two patients who died from the condition were older, immunocompromised, and exhibited poor physical state [4]. For the elderly in poor physical state, the disease quickly progressed to severe cases, which challenged treatment and rehabilitation. Most cases were reported in Lanzhou and Tianshui cities, which could be related to their large number of permanent residents and migrant populations. As such, controlling the disease in the two cities was more challenging.

**Figure 1:**
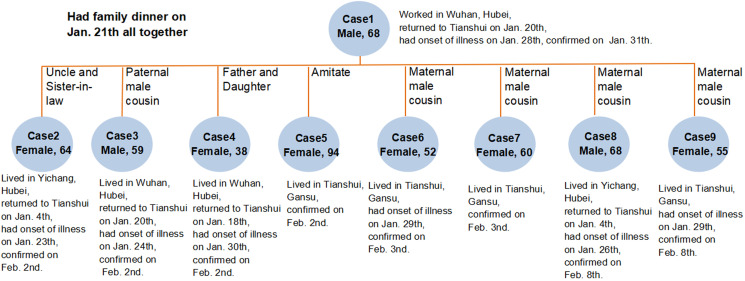
A case of cluster epidemic in Tianshui.

Most of the new cases had been in physical contact with the confirmed cases [5], suggesting that human-to-human transmission was common. Besides, most of the cases were linked to the epidemic in Wuhan [6]. The cases of COVID-19 reported in Gansu province were initially from people who had traveled from other provinces, but later most cases were from the local human-to-human transmission, particularly within or between households [5]. Thus, family interactions were the main sites where COVID-19 was transmitted, and close contact between family members was the primary mode of transmission. Given that the virus is highly transmissible from person to person [7], it was necessary to strengthen the protection consciousness of family members and beware of the occurrence of household cluster epidemics. A typical case is a cluster epidemic caused by a confirmed case sharing a meal with relatives in Tianshui (**Figure 1**).

Most confirmed COVID-19 cases had fever and cough, which were sometimes accompanied by fatigue, expectoration, headache, chest stuffiness, etc. Some patients only exhibited a cough without fever. Therefore, close contact was strictly avoided to cut off the transmission from asymptomatic patients [8].

**Figure:**
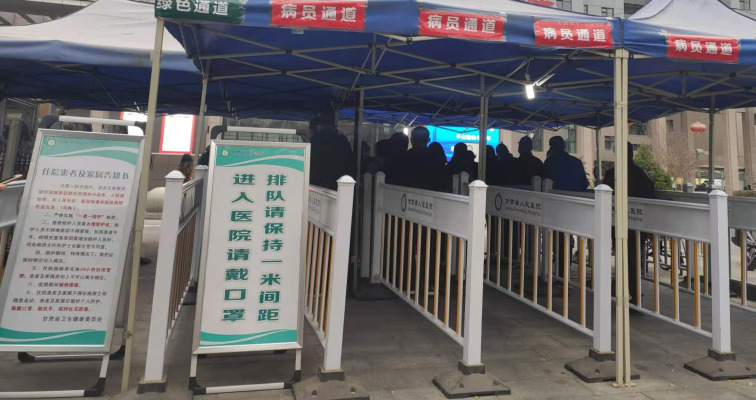
Photo: People queue up to take their temperatures and get into the hospital in Lanzhou, Gansu Province, PR China (from the authors’ own collection, used with permission).

## MEASURES TAKEN TO PREVENT, TREAT, AND CONTROL COVID-19 EPIDEMIC IN GANSU PROVINCE AND THEIR EFFECTS

Gansu province responded quickly following the COVID-19 outbreak. Among the measures taken were early detection and timely isolation and treatment of the patients. Thus, all local cases were cleared in less than two months. Also, the cases from abroad were successively treated within a short time. Gansu province started the first level response of major public health emergencies on January 25, which involved implementing containment measures to curb outbreaks, such as closing schools, cinemas, markets, and other public places. Social gatherings, like holding banquets, parties, and other entertainment, were also stopped. Timely isolation, diagnosis, and treatment of the confirmed cases and their contacts were strictly followed [9]. In total, 91 local cases were confirmed in Gansu province, of which 89 (97.8%) were cured, and 2 (2.2%) died [5], indicating a high survival rate. As of November 3, 2020, no new local case had been reported. It is noteworthy that no case involving a medical staff was reported in Gansu Province, indicating that the first level response of major public health emergencies was efficient.

Moreover, traditional Chinese medicine was employed to treat the condition. Gansu health providers insisted on using a combination of traditional Chinese and Western medicine for the prevention, treatment, and rehabilitation of COVID-19 patients. This leveraged the advantages of traditional Chinese medicine, thereby reducing the mortality rate. As of March 2, 89 (97.8%) cases had been successfully treated using traditional Chinese medicine. Notably, Gansu province ranks high in the use of traditional Chinese medicine for treating COVID-19. The treatment approach has been remarkably effective in treating the disease [10].

In conclusion, the critical measures for preventing and controlling COVID-19 are early detection, timely isolation and treatment of the initial cases, timely contact tracing, and centralized isolation and observation.

## CONCLUSIONS

At present, cases of COVID-19 infections have reduced significantly, however, the disease remains. Besides, with the resumption of daily economic activities, the risk of disease transmission has increased. Therefore, individuals and organizations should still take the necessary precautions to avoid another wave of infections. Epidemic prevention and control is a protracted war and requires concerted efforts of the whole country.
